# Preclinical evaluation of AL-001, a gene therapy for wet age-related macular degeneration

**DOI:** 10.3389/fgene.2026.1836684

**Published:** 2026-09-01

**Authors:** Xuan Liu, Haiyan Liu, Lei Wang, Julia Zhao, Xingwang Xie, Long Jiang, Chunlin Adam Zhao

**Affiliations:** 1 Beijing Tsinghua Changgung Hospital, School of Clinical Medicine, Tsinghua University, Changping, Beijing, China; 2 GCP ClinPlus Co., Ltd., Fengtai, Beijing, China; 3 Anlongbio Inc., Zhongguancun Medical Engineering and Health Industrialization Base, Shunyi, Beijing, China; 4 Beijing CorreGene Biotechnology Co., Ltd, Chang Development Precision Medicine Acceleration Center, Haidian, Beijing, China

**Keywords:** AAV, age-related macular degeneration, AL-001, gene therapy, vascular endothelial growth factor

## Abstract

**Background:**

Frequent intravitreal administration of antivascular endothelial growth factor Vascular endothelial growth factor agents remains a major limitation in the management of wet age-related macular degeneration (wAMD). This study evaluated whether suprachoroidal delivery of an engineered recombinant adeno-associated viral (rAAV)-aflibercept vector could achieve sustained, targeted expression with improved efficacy and safety compared with intravitreal administration.

**Methods:**

AL-001, an engineered rAAV vector expressing aflibercept, was developed and characterized. Its expression profile was first assessed in New Zealand white rabbits following suprachoroidal space (SCS) injection. Efficacy, pharmacokinetics, and safety were then evaluated in a nonhuman primate model of laser-induced choroidal neovascularization (CNV), comparing SCS and intravitreal (IVT) administration routes.

**Results:**

AL-001 efficiently expressed aflibercept in relevant ocular cells *in vitro*. In rabbits, SCS administration produced sustained aflibercept levels in ocular tissues. In the nonhuman primate CNV model, a single SCS injection of AL-001 showed favorable efficacy to IVT injection and a notable mild inflammatory response. At week 4, grade IV lesion incidence was 0% (0/48) after SCS administration versus 14.3% (6/42) after IVT administration (absolute difference, −14.3 percentage points; 95% CI, 3.7%–27.8%; *P* = 0.0258). Throughout follow-up, mean leakage area and grade IV lesion incidence remained 0 with SCS, versus IVT peaks of approximately 0.3 mm^2^ and 33.0%, respectively, declining to 0.03 mm^2^ and 2.0% by day 100. Both the medium and high doses decreased pathological vascular leakage and subretinal hyperreflective material. Vector administration preceded laser-induced CNV modeling, demonstrating that sustained intraocular aflibercept expression in the retina and choroid provided durable antiangiogenic protection. Pharmacokinetic analysis confirmed distinct ocular exposure profiles between routes, with viral genomes confined predominantly to the injected eye and no significant systemic accumulation. AL-001 was well tolerated, without sustained intraocular pressure elevation or severe ocular inflammation, and only mild-to-moderate treatment-emergent adverse events. Low pre-existing anti-AAV2 immunity and time-dependent neutralizing antibody responses postdosing, informing a translational model for patient stratification and redosing feasibility.

**Conclusion:**

Suprachoroidal administration of AL-001 is well tolerated and provides durable, targeted aflibercept expression with pronounced antiangiogenic efficacy. These results support AL-001 as a promising, long-acting therapeutic candidate for wAMD.

## Introduction

Wet age-related macular degeneration (wAMD) is a progressive eye disease that commonly affects people aged 50 years and above (Wong et al., 2014; Apte, 2021). The macula enables detailed central vision. Its degeneration can cause blurred or distorted vision in the central visual field (Zhang et al., 2024). In 10%–15% of AMD patients, the disease progresses to the neovascular (wet) form, which is characterized by choroidal neovascularization (CNV) develops, with abnormal blood vessels growing beneath the macula (Korva-Gurung et al., 2023). This condition is referred to as exudative or wAMD, in which blood and fluid leak from these abnormal vessels into the retina. Fluid accumulation within retinal layers can lead to photoreceptor degeneration, subsequent scar formation, and eventual vision loss (Armendariz and Chakravarthy, 2024).

Vascular endothelial growth factor (VEGF) plays a key role in the development of CNV and its complications, making it an important therapeutic target in disease management. Current clinical treatment for wAMD relies mainly on intravitreal injections of anti-VEGF agents (Corazza et al., 2021; Hang et al., 2023; Li and Huang, 2025). Three recombinant anti-VEGF protein therapies—ranibizumab, bevacizumab, and aflibercept—effectively inhibit VEGF-mediated neovascularization (Sun et al., 2023; Lombardo et al., 2025). These have transformed wAMD treatment and are now the standard of care.

However, a major limitation of current therapies is the need for intravitreal injections every 4–8 weeks (Mitchell et al., 2018). This requires patients to attend regular visits and endure injection discomfort, while also placing logistical burdens on families and caregivers (Yiu et al., 2024). High treatment costs add to the challenge. Long-term follow-up studies show that adherence among wAMD patients is suboptimal, with the actual average injection frequency falling below half of the recommended time interval (Brunner et al., 2025). This often results in disease progression and visual impairment. Moreover, monthly dosing leads to fluctuating exposure levels of anti-VEGF proteins throughout the treatment cycle, creating noticeable peaks and troughs in drug concentration (Rofagha et al., 2013; Wang et al., 2025).

Gene-based delivery of anti-VEGF proteins offers a promising alternative to conventional protein therapy. This approach aims to achieve potent and sustained therapeutic protein expression in the outer retina and choroid, potentially reducing the need for frequent injections (Barthelemy et al., 2025). Gene therapy typically uses viral vectors to deliver genetic material into cells, with recombinant adeno-associated virus (rAAV) being the dominant vector for retinal gene delivery (Campochiaro et al., 2024; Wang et al., 2025). Previous attempts at anti-VEGF gene therapy have employed various vector systems and anti-VEGF transgenes, yet these faced limitations that hindered direct clinical translation (Barthelemy et al., 2025). The rAAV-based therapy, AL-001, has been optimized for suprachoroidal injection and enables efficient protein expression, overcoming previous AAV-related constraints. AL-001 is delivered via a modified adeno-associated virus serotype 2 (AAV2) capsid and carries a potent universal expression cassette containing the codon-optimized cDNA of a recombinant anti-VEGF protein—an already established therapeutic target in wAMD. Therefore, AL-001 has the potential to provide effective intervention before neovascularization leads to vision loss.

Herein, the aim of this study was to evaluate whether suprachoroidal delivery of an engineered rAAV-aflibercept vector could achieve sustained, targeted expression with improved efficacy and safety compared with intravitreal administration.

## Materials and methods

### Vector design and construction

AL-001 is an engineered rAAV vector developed in this study. The capsid is derived from AAV2 and was optimized through site-directed evolution, including the insertion of a short peptide sequence and a single amino acid substitution, resulting in the rAAV514 capsid variant. The expression cassette consists of an optimized tissue-specific promoter, regulatory elements (including TPL2 and eMLP4), a polyadenylation signal, and a codon-optimized aflibercept therapeutic gene. Codon optimization was performed to adjust the GC content to a range favorable for efficient translation in mammalian cells. Vector construction involved capsid engineering and assembly of the expression cassette, and the final vector sequence was verified by Sanger sequencing to confirm sequence accuracy.

### *In Vitro* expression and functional validation

To evaluate the protein expression efficiency of AL-001 in ocular-relevant cell types, Western blot analysis was performed in human retinal pigment epithelial cells (ARPE-19) and human embryonic kidney cells (HEK293) following transduction. Cells were seeded in 6-well plates and transduced with AL-001 at different titers. Total protein was extracted at 72 h post-transduction. The target protein expression was detected using a specific primary antibody followed by a horseradish peroxidase-conjugated secondary antibody. Band intensities were quantified by densitometric analysis using ImageJ software. In parallel, the transduction efficiency was assessed by flow cytometry. Based on comparative protein expression levels and transduction efficiency, AO-1, AO-2, and AO-3 were identified as the top-performing candidate variants for subsequent *in vivo* studies.

### Drug product manufacturing and characterization

The AL-001 drug substance and ophthalmic injectable formulation were comprehensively characterized using analytical methods aligned with the Chinese Pharmacopoeia (2020 edition) and internally validated procedures. The product purity was evaluated by reduced capillary electrophoresis–sodium dodecyl sulfate (CE-SDS) using a noncoated fused-silica capillary, and by size-exclusion high-performance liquid chromatography (SEC-HPLC) performed on a Waters XBridge Protein BEH SEC column to assess protein integrity and aggregation. The proportion of empty and full capsids was determined by anion-exchange HPLC using a CIMac AAV column. The genomic titer was quantified by TaqMan-based digital PCR, while the infectious titer was measured using a 50% tissue culture infectious dose assay in HeLa RC32 cells. The biological potency was assessed through a reporter gene assay in KDR/NFAT-RE HEK293 cells. The particle size distribution and mean hydrodynamic diameter were characterized by dynamic light scattering using an NS-90 nanoparticle analyzer.

The genetic identity of the inserted transgene was confirmed by PCR followed by sequencing of the amplified fragments. Process-related impurities—including residual host cell DNA and proteins, baculovirus-derived components, benzonase, tri-*n*-butyl phosphate, iodixanol, and polysorbate 80—were quantified using validated commercial enzyme-linked immunosorbent assay (ELISA) kits, quantitative PCR assays, or pharmacopeial methods, as appropriate. Product safety was further ensured through testing for replication-competent AAV (rcAAV), bacterial endotoxins, sterility, abnormal toxicity, and microbial limits, in accordance with pharmacopeial requirements.

### Animal models and dosing regimen

New Zealand white rabbits (Suzhou Lakebridge Biotechnology Co., Ltd., China) were used to evaluate the transgene expression kinetics following suprachoroidal space (SCS) administration of AL-001. Cynomolgus monkeys (*Macaca fascicularis*; Guangxi Xiongsen Primate Laboratory Animal Breeding and Development Co., Ltd.) were used to establish a laser-induced CNV model for comparative evaluation of efficacy and safety between the SCS and intravitreal (IVT) administration routes. Animals were randomized into five groups (n = 2–4 per group), including IVT-only, IVT with CNV induction, SCS-only, SCS with CNV induction, and a low-dose group. Group allocation was designed to enable route-specific comparison of efficacy under CNV challenge while simultaneously evaluating dose-dependent ocular safety.

Animals were housed under controlled temperature conditions with *ad libitum* access to food and water. AL-001 was administered as a single 50-µL injection via either the SCS or IVT route at doses of 2.0 × 10^13^ vg/mL (high dose) and 1.0 × 10^13^ vg/mL (low dose). The selection of dose levels and administration routes was based on prior internal studies and preclinical optimization experiments. SCS injections were performed using a microneedle-based delivery system with a depth-limiting mechanism, inserted 2–3 mm posterior to the limbus. Correct placement within the SCS was confirmed by optical coherence tomography (OCT) following injection. IVT injections were performed at a site approximately 2–3 mm posterior to the limbus, while suprachoroidal injections were administered using a 38G microneedle with controlled depth to ensure delivery into the suprachoroidal space. All animal procedures were conducted in accordance with the Association for Research in Vision and Ophthalmology Statement on the Use of Animals in Ophthalmic and Vision Research and complied with Association for Assessment and Accreditation of Laboratory Animal Care guidelines. Experimental protocols were approved by the Institutional Animal Care and Use Committee, and animal numbers were minimized in accordance with the principles of replacement, reduction, and refinement.

A laser-induced CNV model was established 36 days after vector administration, as previously described (Lambert et al., 2013; Gong et al., 2015). Based on prior *in vitro* and *in vivo* characterization of AAV vectors, transgene expression is typically initiated within 48 h and reaches a relatively stable level within 96 h after administration. Accordingly, the timing of downstream efficacy evaluation and CNV induction was selected to ensure sufficient transgene expression prior to model establishment. Bilateral fundus laser photocoagulation was performed to create 6–8 burns per eye using a 532-nm laser (400–500 mW, 50-µm spot size, 100-ms duration). Laser spots were applied in the posterior pole region while avoiding major retinal vessels. Postprocedural care included topical antibiotic ointment. Animal health was monitored through twice-daily general observations and detailed weekly examinations.

Comprehensive ophthalmic evaluations were conducted under mydriasis and anesthesia in a standardized sequence, including general examination, dark- and light-adapted electroretinography, intraocular pressure (IOP) measurement, OCT, fundus photography, and fundus fluorescein angiography (FFA). For FFA, sodium fluorescein was injected intravenously, and leakage from the laser lesions was graded on a four-point scale (grade 1, no leakage, faint or mottled hyperfluorescence without progressive intensification or expansion; grade 2, suspected leakage, localized hyperfluorescence without progressive increase in size or intensity; grade 3, definite leakage, hyperfluorescence shows increased intensity but no enlargement of boundaries; grade 4, pathological leakage, hyperfluorescence shows progressive increase in both size and intensity with fluorescein pooling beyond the lesion border). The primary efficacy endpoints were the incidence of grade 4 lesions and the mean leakage area. OCT was used to measure the thickness of the subretinal hyperreflective material.

### Sample collection and processing

At predefined time points, aqueous humor (∼100 µL), peripheral blood (3 mL, EDTA-anticoagulated), and ocular tissues, including the cornea, retina, and choroid, were collected. Ocular tissues were isolated according to anatomical structure without volumetric quantification, and downstream assay input was normalized using validated protocol-specific procedures. Selected samples were immediately snap-frozen on dry ice and stored at −60 °C to −90 °C until analysis.

Nonocular tissues (∼20–50 mg each), including the heart, liver, spleen, kidney, brain regions, and gonads, were collected posteuthanasia and either snap-frozen or preserved in RNAlater solution. Plasma was separated within 1 h after blood collection and stored for subsequent pharmacokinetic and immunogenicity analyses.

### Pharmacokinetic and biodistribution analyses

Viral genome copy numbers were quantified in both ocular and systemic tissues using quantitative polymerase chain reaction (qPCR) with specific primers targeting the inverted terminal repeat region of the AAV vector. Absolute quantification was performed using a standard curve methodology. Therapeutic protein (aflibercept) concentrations in ocular fluids (aqueous and vitreous humor), ocular tissues (retina and choroid), and plasma were measured using validated ELISA kits from the Quantikine series (R&D Systems). For plasma analysis, peripheral blood was collected in EDTA-anticoagulated tubes, and plasma was separated within 1 h after collection and stored at −60 °C to −90 °C until analysis.

### Histological and molecular analysis

Tissue samples were fixed in 4% paraformaldehyde, embedded in paraffin, sectioned, and stained with hematoxylin and eosin for histopathological evaluation by light microscopy.

Genomic DNA and total RNA were extracted from tissue samples (∼20–50 mg) using the MagMAX™ DNA Multi-Sample Ultra 2.0 kit and MagMAX™ mirVana™ Total RNA Isolation Kit (Applied Biosystems, Thermo Fisher Scientific, Waltham, MA, United States) according to the manufacturer’s instructions. RNA samples were preserved in RNAlater prior to extraction. Quantification of viral genome DNA and transgene mRNA expression was performed using validated qPCR and RT-qPCR assays, respectively (Hays et al., 2024).

Expression of the target protein in ocular tissues was assessed by immunofluorescence staining using a specific primary antibody followed by a fluorescently labeled secondary antibody. Viral genome copy numbers were quantified by qPCR using primers targeting the inverted terminal repeat region of the vector. Absolute quantification was performed using standard curve methodology.

Target protein concentrations were measured using ELISA kits from the Quantikine series (R&D Systems), according to the manufacturer’s instructions.

### Immunogenicity assessment

Serum anti-AAV2 neutralizing antibody (NAb) titers were measured using a reporter gene-based assay utilizing HEK293 cells and an AAV2 vector encoding a luciferase reporter gene. Serially diluted serum samples were co-incubated with the reporter virus, and inhibition of luciferase activity was measured. The NAb titer was defined as the reciprocal of the highest serum dilution resulting in ≥50% inhibition of reporter gene expression compared with antibody-free controls.

### Clinical study design and safety assessment

The AL-001–01 study was a prospective, multicenter, open-label, nonrandomized, single-arm, dose-escalation phase I/IIa clinical trial designed to evaluate the safety, tolerability, and pharmacokinetics of AL-001 in patients with wAMD. The clinical component was included as an exploratory translational safety assessment and was not designed or powered to evaluate clinical efficacy outcomes. The study was conducted at Peking Union Medical College Hospital and Tianjin Medical University Eye Hospital in China. Eligible patients were aged 50–75 years old and had advanced wAMD with subfoveal CNV in the study eye. All participants had previously received intravitreal aflibercept and demonstrated a documented anatomical response to anti-VEGF therapy. Best-corrected visual acuity (BCVA) at screening was required to be 19–63 Early Treatment Diabetic Retinopathy Study (ETDRS) letters in the dose-escalation cohorts and 31–73 ETDRS letters in the expansion cohort. Key exclusion criteria included ocular conditions affecting central vision other than wAMD, significant prior ocular surgery in the study eye, active intraocular inflammation or infection, severe uncontrolled systemic disease, and known immunological disorders.

AL-001 was administered as a single injection into the SCS using a dedicated micro-injector (Beijing Medical Robots Industry Innovation Center, Beijing, China) equipped with a 38-gauge microneedle (900 µm in length) and a depth-limiting mechanism to ensure targeted SCS delivery. The procedure was performed under topical local anesthesia in an outpatient setting. The injection volume was 100 μL per eye. A standard 3 + 3 dose-escalation design was employed with three planned dose levels: 1 × 10^11^, 3 × 10^11^, and 1 × 10^12^ vector genomes per eye. All enrolled subjects received a single intravitreal injection of aflibercept (2 mg) in the study eye on day 1. Eligibility was reconfirmed at week 1 based on predefined anatomical response criteria. Subjects meeting these criteria received a single SCS injection of AL-001 at week 2.

Safety evaluations included both ocular and systemic assessments throughout the 24-week follow-up period. Ocular assessments comprised BCVA (ETDRS protocol), IOP measurement, slit-lamp biomicroscopy, dilated fundus examination, OCT, and FFA, as clinically indicated. Systemic safety evaluations included vital signs, physical examination, clinical laboratory testing, electrocardiography, echocardiography, and documentation of concomitant medications. Adverse events (AEs) were recorded and graded according to the National Cancer Institute Common Terminology Criteria for Adverse Events, version 5.0. Investigators assessed the relationship of each AE to the study drug and/or procedure. While the study protocol required comprehensive monitoring of all AEs and serious AEs as primary safety endpoints, the safety analysis focused on treatment-emergent adverse events (TEAEs)—defined as events that emerged or worsened after administration. This approach enabled a more precise characterization of the clinical safety profile directly attributable to AL-001.

### Ocular inflammation scoring

Ocular inflammation was assessed by slit-lamp biomicroscopy using a standardized grading system adapted from the modified Hackett–McDonald scale and the Standardization of Uveitis Nomenclature (SUN) criteria. The average inflammation score was calculated as a composite of conjunctival hyperemia (0–3), corneal opacity (0–4), and iritis (0–4), based on the extent and severity of visible clinical signs. In addition, intraocular inflammation parameters, including anterior chamber cells and vitreous opacities, were evaluated using a semi-quantitative grading scale (0 to ++++), based on the extent of cellular infiltration and opacity observed.

### Ethics statement

All animal procedures were performed in accordance with the ARVO Statement for the Use of Animals in Ophthalmic and Vision Research and were approved by the Institutional Animal Care and Use Committee of JOINN Laboratories (Suzhou) Co., Ltd. (Approval No.: Q20-S354-PK).

The studies involving humans were approved by Ethics Committee of Peking Union Medical College Hospital, Chinese Academy of Medical Sciences (Approval No.: HS2023035). The studies were conducted in accordance with the local legislation and institutional requirements. The participants provided their written informed consent to participate in this study. The animal study was approved by Institutional Animal Care and Use Committee of JOINN Laboratories (Suzhou) Co., Ltd. (Approval No.: Q20-S354-PK). The study was conducted in accordance with the local legislation and institutional requirements. No potentially identifiable images or data are presented in this study.

The clinical study was conducted in compliance with the Declaration of Helsinki and Good Clinical Practice guidelines. The study protocol was approved by the Ethics Committee of Peking Union Medical College Hospital, Chinese Academy of Medical Sciences (Approval No.: HS2023035), and written informed consent was obtained from all participants prior to enrollment.

The trial was prospectively registered at Chinese Clinical Trial Registry (https://www.chictr.org.cn/, Identifier: ChiCTR2300074365).

### Statistical analysis

All statistical analyses were performed using SPSS version 13.0 (IBM, USA). Categorical variables were analyzed using Fisher’s exact test. Continuous variables were assessed for normality and analyzed using parametric tests (one-way analysis of variance [ANOVA]) or nonparametric tests (Mann–Whitney U or Kruskal–Wallis tests), as appropriate. When the ANOVA results were statistically significant, post hoc comparisons were performed using the least significant difference test. A two-sided *P*<0.05 was considered statistically significant.

## Results

### Molecular design and *in vitro* protein expression of AL-001

**FIGURE 1:**
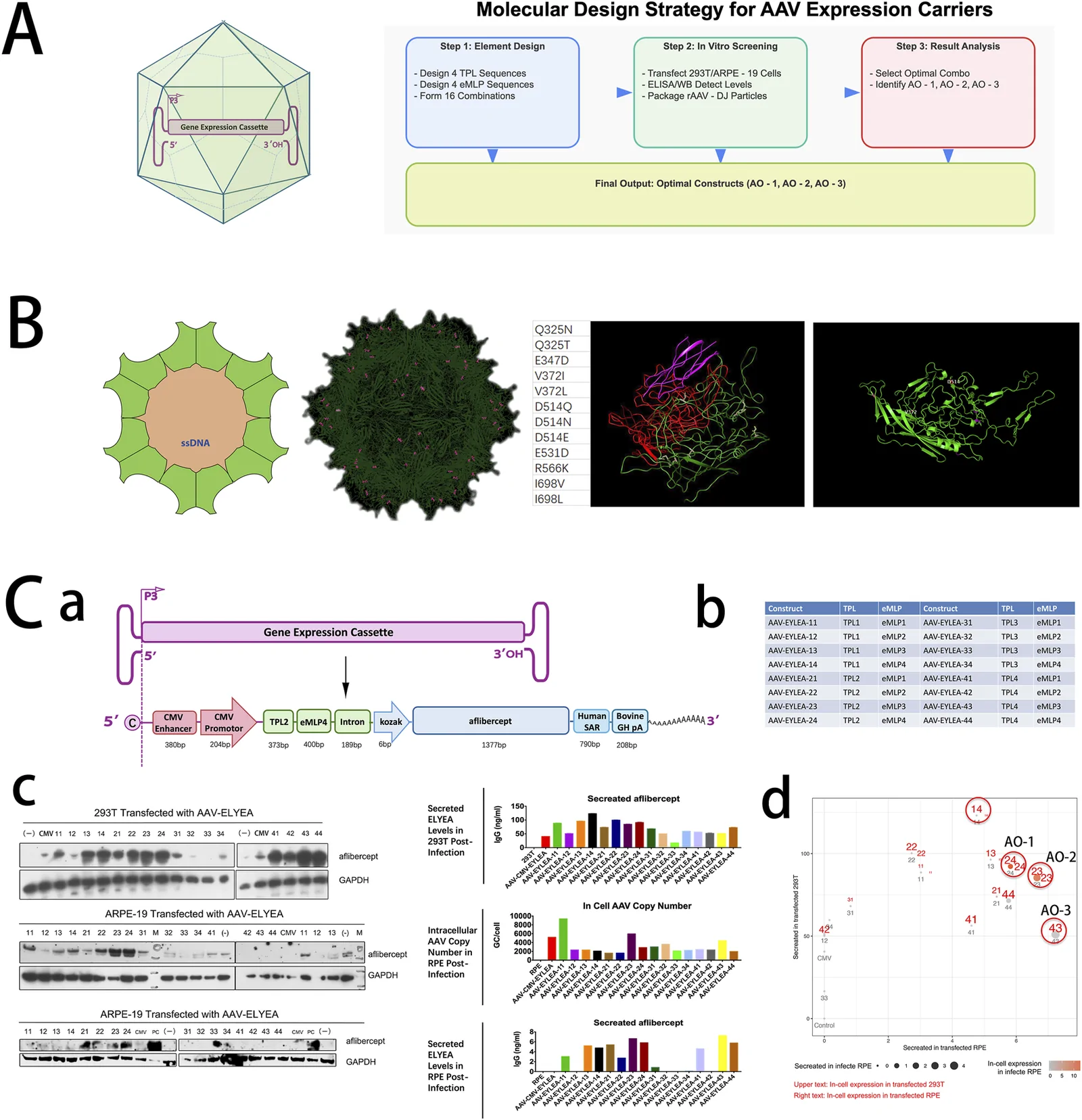
*In vitro* characterization of AL-001 **(Aa)** Schematic of the engineered recombinant AAV vector AL-001 **(Ab)** Workflow for the molecular design and screening process **(Ba)** Structural diagram of the capsid protein of AAV2 **(Bb)** Three-dimensional protein structure simulation highlighting specific amino acid mutations (e.g., Q325N, V372I, etc.), with a corresponding list of modification sites **(Ca)** Linear map of the gene expression cassette encoding aflibercept, featuring a CMV enhancer/promoter, specific translational enhancers (TPL) and promoter elements (eMLP), a chimeric intron, kozak sequence, human scaffold attachment region (SAR), and bovine GH poly(A) signal **(Cb)** Table listing the matrix of 16 construct variants (AAV-EYLEA-11 to −44) generated by combining different TPL and eMLP sequences (Cc) Western blot analysis and quantification of the target protein in retinal ARPE-19 cells and HEK293 cells **(Cd)** Quantitative evaluation of the AAV infection model in ARPE-19 cells.

The recombinant adeno-associated virus vector AL-001 that incorporates an engineered therapeutic gene expression cassette based on an optimized AAV2-derived capsid was successfully constructed (Figures 1Aa). The overall vector development strategy included capsid engineering and expression cassette optimization (Figures 1Ab).

To enhance transduction efficiency in intraocular target cells, site-directed evolution was applied to the AAV2 capsid. Specific point mutations were introduced at selected positions, and three-dimensional structural modeling confirmed that these modifications preserved capsid integrity while altering surface-exposed regions (Figures 1Ba,b).

The expression cassette was further optimized to achieve sustained protein expression in ocular cells. It contains a tissue-specific promoter, optimized regulatory elements (including TPL2 and eMLP4), a polyadenylation signal, and a codon-optimized therapeutic gene. Codon optimization adjusted the GC content to a range favorable for efficient translation in mammalian cells (Figures 1Ca,b).

*In vitro* protein expression was evaluated by Western blot analysis. Detectable target protein expression was observed in ARPE-19 cells following AL-001 transduction (Figures 1Cc). Quantitative analysis of the transduction efficiency in ARPE-19 cells further identified AO-1, AO-2, and AO-3 as the top-performing lead candidates among the tested variants (Figures 1Cd).

Together, these preliminary findings suggest that AL-001 is capable of mediating detectable transgene expression in ARPE-19 cells under *in vitro* conditions, supporting its further evaluation for subsequent *in vivo* evaluation.

### AL-001 drug substance characterization, integrity, and *in vivo* expression

**FIGURE 2:**
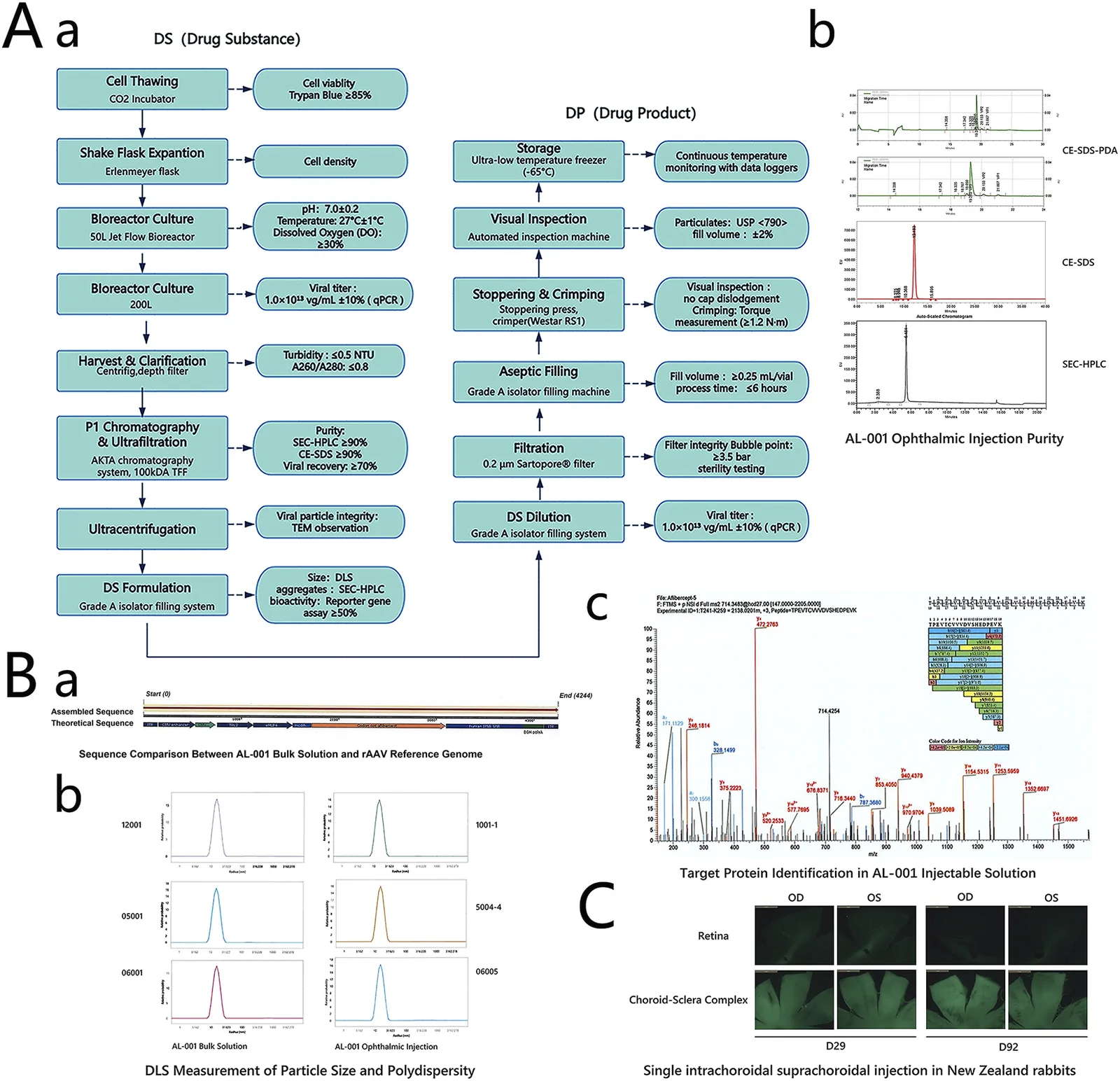
Manufacturing process and quality control of AL-001 **(Aa)** Schematic illustration of the standardized production workflow for the AL-001 drug substance **(Ab)** Analytical data of HPLC chromatograms **(Ba)** Deep sequencing analysis of the capsid gene and the packaged therapeutic expression cassette. The target protein was successfully identified in the final drug product formulation **(Bb)** Dynamic light scattering measurement of the particle size distribution and polydispersity **(Bc)** Mass spectrometry peptide mapping identifying the target protein sequence in the injectable solution **(C)** Retinal immunofluorescence staining in New Zealand white rabbits. OD: right eye; OS: left eye; D29, Day 29; D92, Day 92.

The AL-001 drug substance and ophthalmic drug product were successfully manufactured and exhibited consistent quality attributes throughout the production and formulation processes (Figure 2A). Analytical characterization demonstrated high purity of the final injectable product, as evidenced by the CE-SDS and SEC-HPLC profiles showing effective removal of process- and product-related impurities (Figures 2Ab). Furthermore, rigorous controls for exogenous agents and viral clearance were implemented throughout manufacturing. The methods and corresponding acceptance criteria for *mycoplasma*, endotoxin, residual DNA, and adventitious virus testing are summarized in Table 1, ensuring the safety profile of the drug substance.

**TABLE 1:** Summary of exogenous factor control, virus clearance methods, and acceptance criteria.

Control stage	Test item	Method	Acceptance criteria
Bac-514 working virus, Вас-AО2 working virus	*Mycoplasma* test	qPCR method	Should be negative [5]
	Bacterial endotoxin test	Gel clot method	Should be less than 20 EU/mL [5]
Control cells	Exogenous viral factor test	Direct observation of Sf9 cells	No cytopathic effect observed; should be negative [5]
	Human, monkey, or homologous cell culture method	No cytopathic effect observed	No cytopathic effect observed; should be negative [5]
	Hemadsorption virus test	All cells inoculated with test samples	Should be negative [5]
Harvest fluid	*Mycoplasma* test	qPCR method	Should be negative [5]
	Bacterial endotoxin test	Gel clot method	Should be less than 20 EU/mL [5]
Bulk solution	Residual baculovirus DNA	qPCR method	Should not exceed 100.0 ng/1.00 × 10^12^ vg [5]
	Residual Sf9 cell DNA	qPCR method	Should not exceed 10.0 ng/1.00 × 10^12^ vg [5]
	Residual baculovirus protein	ELISA method	Should not exceed 50.0 ng/mL [5]
	Residual Sf9 cell protein	ELISA method	Should not exceed 200.0 ng/mL [5]
	Residual baculovirus particles	Plaque assay	No plaques should be observed [5]
Formulation	Bacterial endotoxin test	Gel clot method	Should be less than 2.0 EU/mL [5]
	Microbial limit	Membrane filtration method	Should not exceed 3 CFU/10 mL [5]
	rcAAV	Cell culture method	Should be less than 1 rcAAV/1 × 10^8^ vg [5]
	Bacterial endotoxin test	Gel clot method	Should be less than 2.0 EU/mL [5]
	Sterility test	Membrane filtration method	Should meet requirements [5]
	Abnormal toxicity	Mouse and Guinea pig method	Should meet requirements

ELISA, enzyme-linked immunosorbent assay; qPCR, quantitative polymerase chain reaction; vg, viral genomes.

The genetic integrity of the purified viral particles was further confirmed by deep sequencing analysis. Sequence alignment revealed complete concordance between the AL-001 bulk solution and the reference rAAV genome, with no unintended mutations or recombination events detected across the capsid gene and the packaged therapeutic expression cassette, indicating high genetic stability (Figures 2Ba). In addition, dynamic light scattering analysis demonstrated a narrow particle size distribution and low polydispersity for both the AL-001 bulk solution and the ophthalmic injection, consistent with a uniform viral particle population (Figures 2Bb).

To further evaluate the storage stability of the AL-001 formulation, we conducted a long-term stability study under conditions of −20 °C ± 5 °C. As shown in Table 2, the formulation maintained a clear appearance, stable genomic titer, and satisfactory purity parameters over 6 months. However, a decline in biological activity was observed starting from the third month, indicating the need for further optimization of formulation protection strategies in the future. It is important to note that these results at −20 °C represent an early-stage formulation exploration designed to characterize the vector’s sensitivity under various stress conditions.

**TABLE 2:** Stability summary of AL-001 ophthalmic solution (−20 °C ± 5 °C long-term storage).

Time point	Appearance	Genome titer (viral genome copies/mL)	Biological activity (%)	Purity (CE-SDS)	Purity (SEC-HPLC)	Purity (AEX-HPLC)	Particle size (NM)
0 months	Clear colorless liquid	1.20 × 10^13^	97%	94.8% (VP3 clip: 4.5%)	98.00%	94.97% empty/95.03% solid	29.8 (100%)
1 month	Clear colorless liquid	1.22 × 10^13^	97%	95.0% (VP3 clip: 4.3%)	98.40%	95.46% empty/94.54% solid	29.9 (100%)
3 months	Clear colorless liquid	1.01 × 10^13^	53%	94.9% (VP3 clip: 4.4%)	98.40%	95.87% empty/94.13% solid	30.1 (100%)
6 months	Clear colorless liquid	9.36 × 10^12^	45%	94.6% (VP3 clip: 4.3%)	99.00%	96.36% empty/93.64% solid	29.4 (100%)

CE-SDS, capillary electrophoresis–sodium dodecyl sulfate; SEC-HPLC, size-exclusion high-performance liquid chromatography; AEX-HPLC, anion-exchange HPLC.

Mass spectrometry analysis of the AL-001 injectable solution successfully identified the target therapeutic protein, confirming correct protein expression and molecular integrity in the final drug product (Figures 2Bc). To assess functional expression *in vivo*, immunofluorescence staining was performed on retinal and choroid–sclera complex sections from New Zealand white rabbits following a single suprachoroidal injection. Detectable target protein signal was observed in ocular tissues following suprachoroidal administration, however, due to the absence of corresponding control images, these findings should be interpreted with caution and are presented as providing qualitative evidence of transgene expression (Figure 2C).

### AL-001 reduces CNV-associated vascular leakage

**FIGURE 3:**
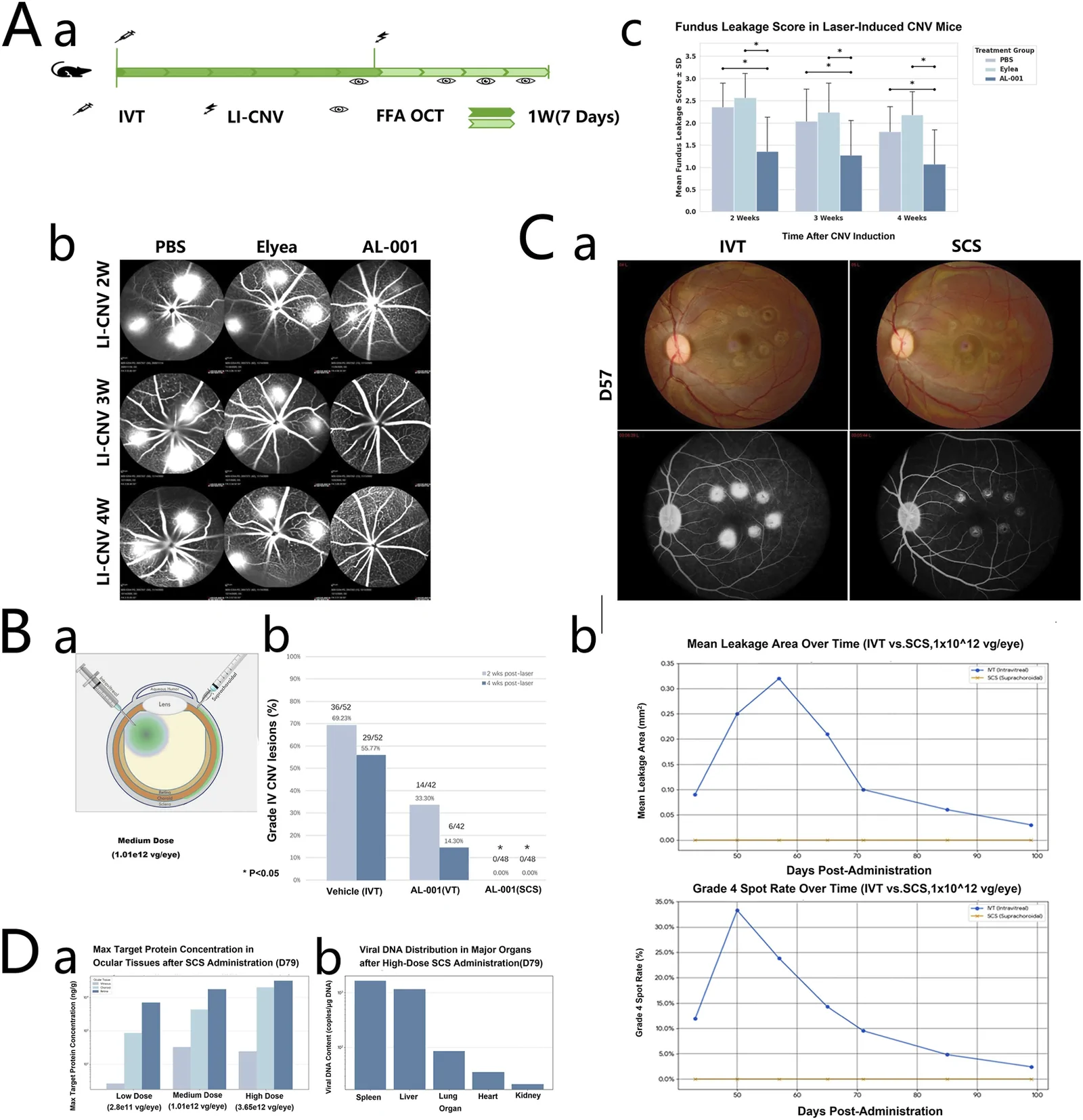
AL-001 function in a laser-induced choroidal neovascularization (CNV) model **(Aa)** Schematic timeline of the experimental design, including laser induction, intravitreal (IVT) injection, and weekly follow-ups using fundus fluorescein angiography (FFA) and optical coherence tomography **(Ab)** Representative FFA images at 2, 3, and 4 weeks postinjury. Treatment groups include phosphate-buffered saline (PBS; vehicle), Eylea (positive control), and AL-001 **(Ac)** Quantitative analysis of the mean fundus leakage scores. AL-001 treatment significantly reduced vascular leakage compared to PBS, demonstrating efficacy comparable to the standard-of-care, Eylea (n = 7–11 animals/group). Group comparisons were performed using ANOVA with LSD post-hoc test. *P < 0.05 was considered statistically significant **(Ba)** Schematic diagram of IVT and suprachoroidal space (SCS) injections **(Bb)** Percentage of grade IV CNV lesions observed at 2 and 4 weeks postlaser **(Ca)** Representative fundus photography and angiography at day 57 comparing IVT and SCS delivery of AL-001 (1 × 10^12^ viral genomes/eye) **(Cb)** Time-course quantification of the mean leakage area (top) and the grade-4 spot rate (bottom) **(Da)** Pharmacokinetic profile of the target protein concentration in ocular fluids after IVT versus SCS administration (n = 4 animals/group) **(Db)** Biodistribution of viral vector DNA in major organs (spleen, liver, lung, heart, kidney) on day 79 (n = 4 animals/group).

In the laser-induced choroidal neovascularization mouse model, AL-001 was administered via IVT, and disease progression was evaluated using FFA and OCT (Figures 3Aa). Representative FFA images showed reduced fluorescein leakage in AL-001-treated eyes compared with vehicle controls at 2, 3, and 4 weeks after CNV induction (Figures 3Ab). Quantitative analysis showed that the AL-001 group exhibited consistently lower leakage scores across all examined time points (Figures 3Ac), decreasing from 1.4 ± 0.8 at week 2 to 1.1 ± 0.8 at week 4, compared with 2.4 ± 0.5 and 1.8 ± 0.6 in the PBS group and 2.6 ± 0.6 and 2.2 ± 0.5 in the Eylea group.

To compare the ocular delivery routes, a schematic diagram illustrates the drug distribution following IVT and SCS administration (Figures 3Ba). The CNV lesions were graded based on the FFA leakage severity. At week 4 postadministration, the proportion of grade IV leakage lesions was significantly lower in the SCS group (0.0%, 0/48) than in the IVT group (14.3%, 6/42; 95% CI, 3.7%–27.8%; *P* = 0.0258), while the vehicle group (55.8%, 29/52, *P* < 0.0001) (Figures 3Bb).

Longitudinal analysis further showed that SCS administration induced sustained suppression of CNV leakage compared with IVT during the observation period (Figures 3Ca,b), maintaining both mean leakage area and grade IV lesion incidence at 0, whereas the IVT group showed peak values of ∼0.3 mm^2^ and ∼33.0%, respectively, which declined to ∼0.03 mm^2^ and ∼2.0% by day 100. Pharmacokinetic analysis revealed distinct intraocular exposure profiles following IVT and SCS administration, as determined by the target protein concentrations in the aqueous humor and vitreous (Figures 3Da). As summarized in Table 3. SCS administration resulted in dramatically higher drug exposure than IVT administration in the vitreous (127 ± 70 vs. 7 ± 11 ng/mL, *P* < 0.05), retina (10,805 ± 8,916 vs. 15 ± 9 ng/g, *P* < 0.001), and choroid/sclera complex (2,393 ± 1,584 vs. 34 ± 35 ng/g, *P* < 0.01). Conversely, aqueous humor concentrations were relatively low between the two routes (38 ± 18 ng/mL for SCS vs 22 ± 30 ng/mL for IVT, *P* < 0.05), confirming the excellent posterior targeting profile of SCS with restricted anterior segment exposure. Biodistribution analysis demonstrated that the viral genome copies were predominantly detected in the injected eye, with no detectable accumulation in the major peripheral organs (Figures 3Db).

**TABLE 3:** Comparison of ocular tissue drug concentrations following SCS versus IVT administration.

Tissue type	IVT	SCS	*P*
Vitreous (ng/mL)	7 ± 11 (range: 1.0–33.3, n = 9)	127 ± 70 (range: 71.2–289, n = 10)	<0.05
Retina (ng/g)	15 ± 9 (range: 5.90–26.2, n = 6)	10,805 ± 8,916 (range: 23.4–28,250, n = 12)	<0.001
Choroid/Sclera complex (ng/g)	34 ± 35 (range: 4.52–87.5, n = 5)	2,393 ± 1,584 (range: 675–5,050, n = 10)	<0.01
Aqueous humor (ng/mL)	22 ± 30 (range: 2.68–75.2, n = 5)	38 ± 18 (range: 18.4–65.9, n = 12)	<0.05

IVT, intravitreal; SCS, suprachoroidal space.

1 Statistical significance was determined using the Mann-Whitney U test between IVT, and SCS, groups.

### Safety of AL-001 injection

**FIGURE 4:**
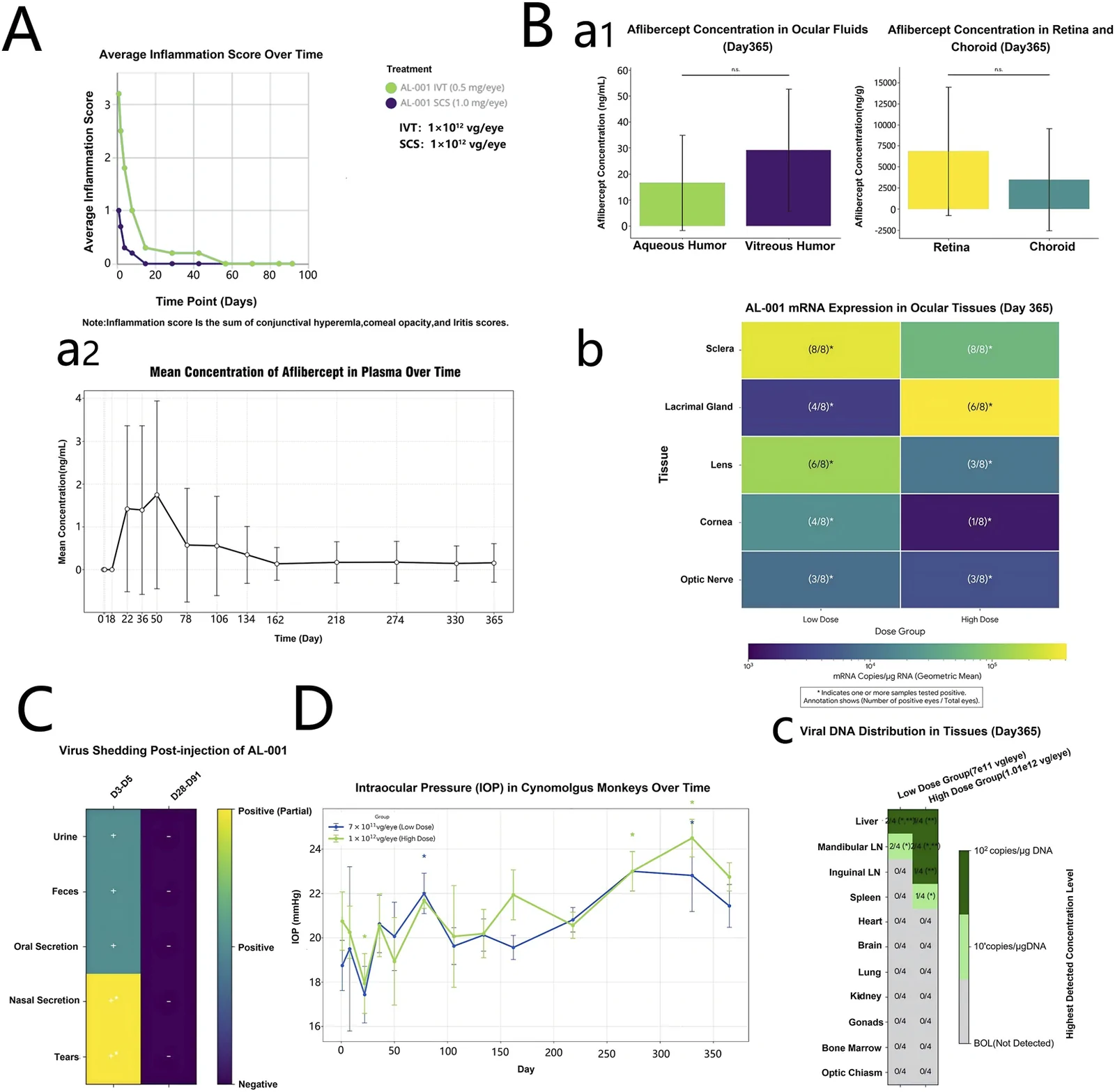
Local tolerability and systemic safety profile of AL-001 **(A)** Assessment of intraocular inflammation in macaques following suprachoroidal space (SCS) injection and intravitreal (IVT) injection **(Ba1)** Concentration of aflibercept protein in ocular fluids (aqueous humor, vitreous humor) and tissues (retina, choroid). Data are presented as mean±SD (n = 4 animals/group). Group comparisons were performed using independent-samples t-test. Ns, not significant **(Ba2)** Systemic pharmacokinetics: Mean plasma concentration of aflibercept over a 365-day period (n = 4 animals/group) **(Bb)** Heatmap of AL-001 mRNA expression copies across the anterior and posterior ocular tissues (sclera, lacrimal gland, lens, cornea, optic nerve) **(Bc)** The viral DNA content was quantified in various tissues and organs. LN: lymph nodes **(C)** Viral clearance postadministration of AL-001 (n = 5, 10 eyes/group) **(D)** Intraocular pressure (IOP) measurements before and after a single AL-001 injection. Data are presented as mean±SD (n = 4, 8 eyes/group). Group comparisons were performed using a Linear Mixed-Effects Model (LMM) accounting for repeated measures and intra-subject eye correlation, followed by Bonferroni’s post-hoc test. *P < 0.05 was considered statistically significant.

The average inflammation score decreased after dosing in both groups and remained low throughout the observation period. The IVT group showed a peak score of 3.2 ± 0.8 at day 0 that gradually declined to 0 by day 60, whereas the SCS group peaked at only 1.0 ± 0.0 and reached 0 by day 15, remaining undetectable thereafter. Overall, the inflammation scores were lower in the SCS group compared with the IVT group (Figure 4A).

Ocular exposure of the expressed therapeutic protein aflibercept was assessed on day 365. Aflibercept concentrations were detected in the aqueous humor, vitreous humor, and retina/choroid following AL-001 administration (Figures 4Ba). The plasma aflibercept concentrations remained low throughout follow-up, rising transiently to a peak of 1.7 ± 2.2 ng/mL at day 50 before declining to 0.6 ± 1.3 ng/mL by day 78 and stabilizing at 0.2 ± 0.5 ng/mL through day 365, with no evidence of sustained exposure (Figures 4Ba).

In parallel, RT–qPCR analysis demonstrated detectable AL-001 mRNA expression in multiple ocular tissues on day 365, with detectable mRNA expression observed across multiple ocular tissues, with variability between dose groups (Figures 4Bb).

Biodistribution analysis showed that viral DNA was primarily detected in ocular tissues, while limited viral DNA copies were detected in select nonocular tissues on day 365, and no viral DNA was detected in most peripheral organs (Figures 4Bc). Consistent with this finding, viral genome copies in the peripheral blood were transiently detectable after administration and declined to below the limit of detection during follow-up (Figure 4C).

IOP was monitored at predefined time points following AL-001 administration. The IVT group maintained relatively stable IOP values (15.5 ± 0.9 to 16.3 ± 1.3 mmHg), whereas the SCS group ranged from 17.5 ± 2.8 to 22.3 ± 2.1 mmHg. Significant differences between the two groups were observed at most post-treatment time points (all *P* < 0.01), except for day 8 (*P* = 0.237) (Figure 4D).

### Immunogenicity assessment and translational modeling

**FIGURE 5:**
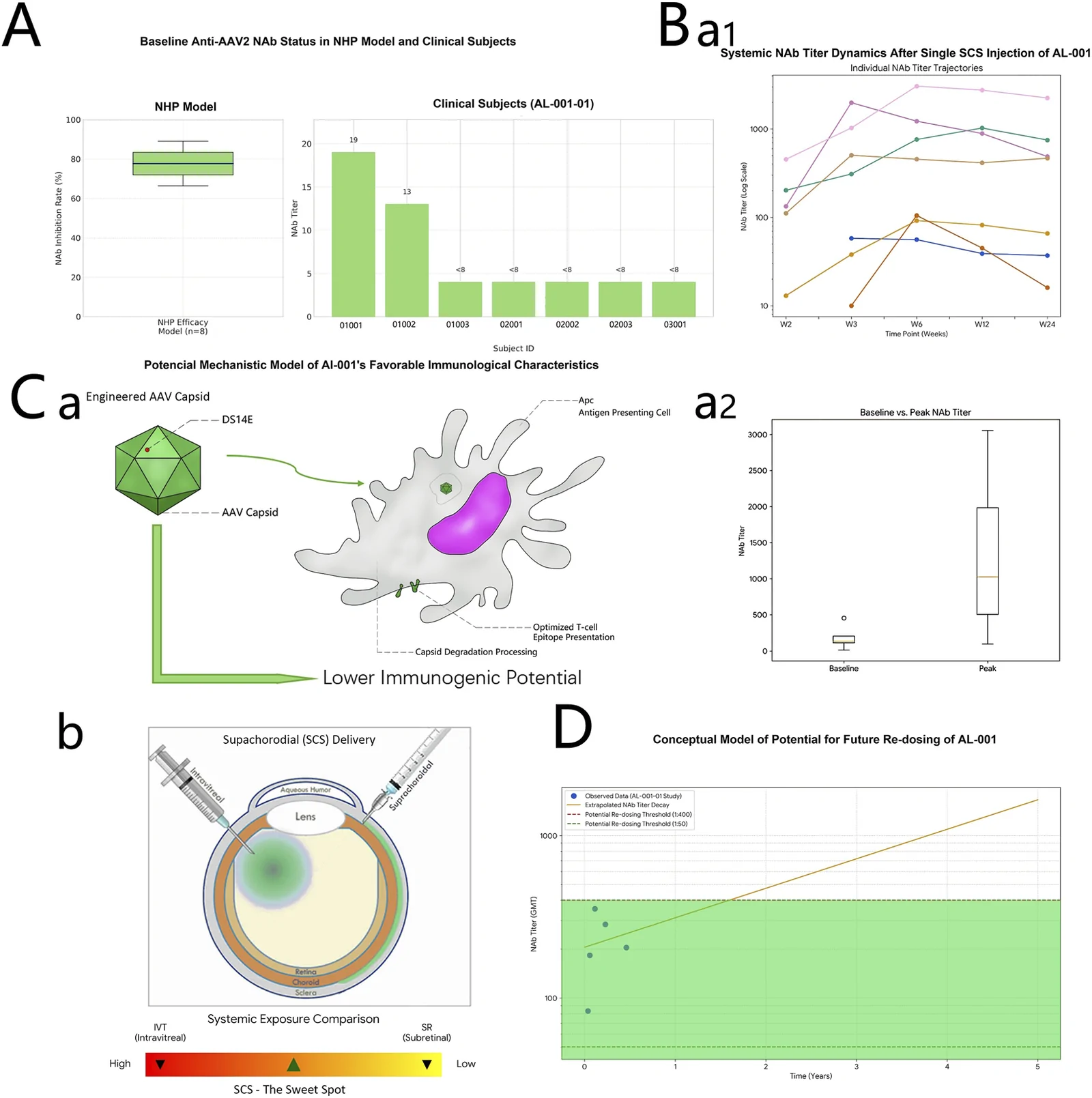
Immunogenicity analysis and clinical translation model **(A)** Distribution of baseline serum neutralizing antibody (NAb) titers against wild-type AAV2 and the engineered AL-001 capsid in nonhuman primates (n = 8) and clinical subjects (n = 7) **(Ba1)** Individual longitudinal trajectories of NAb titers (log scale) over 14 weeks postinjection **(Ba2)** Comparison of baseline vs. peak NAb titers **(Ca)** Schematic illustration showing the engineered AAV capsid (DS14E) and its interaction with an antigen-presenting cell (APC) **(Cb)** Diagram comparing systemic exposure following different ocular delivery routes **(D)** Conceptual model evaluating the feasibility of future redosing. The plot shows the observed NAb titer data from the AL-001–01 study (blue dots) and a projected decay curve (orange line) based on a linear extrapolation model. The green shaded area represents the potential therapeutic window for redosing, suggesting that the NAb titers may fall back to permissible levels over time (e.g., after 2–3 years), allowing for a second administration if needed.

Immunogenicity assessment and translational modeling baseline anti-AAV2 NAb levels were evaluated in the nonhuman primate model and in clinical subjects. The majority of nonhuman primates (78.0%) and enrolled subjects (71.4%) exhibited baseline NAb titers below the predefined positivity threshold (Figure 5A). Following AL-001 administration, longitudinal monitoring revealed a time-dependent increase in serum NAb titers in cynomolgus monkeys. The NAb levels began to rise at approximately 2–4 weeks after dosing, reached individual peak titers ranging from ∼60 to ∼4,000 between weeks 3 and 12, and subsequently showed stabilization or decline during follow-up (Figures 5Ba). Comparison of the baseline and peak titers further demonstrated inter-individual variability in the magnitude of the humoral immune response (Figures 5Ba; Figure 4D). Based on the observed baseline NAb distribution and postadministration NAb dynamics, a data-informed conceptual framework was constructed to support clinical translation. This framework integrates the baseline immune status and longitudinal NAb kinetics as variables potentially associated with treatment durability and clinical outcomes, while acknowledging variability across subjects (Figures 5Ca,b). In addition, a conceptual redosing model was generated by integrating the observed NAb kinetics with the projected antibody decay over time. Systemic NAb titers peaked at a GMT of ∼347 at week 6 and declined to ∼204 by week 24, with further projected decay below the predefined 1:400 and 1:50 thresholds, supporting a potential temporal window for future redosing evaluation (Figure 5D).

### SCS delivery approach and clinical safety profile of AL-001

**FIGURE 6:**
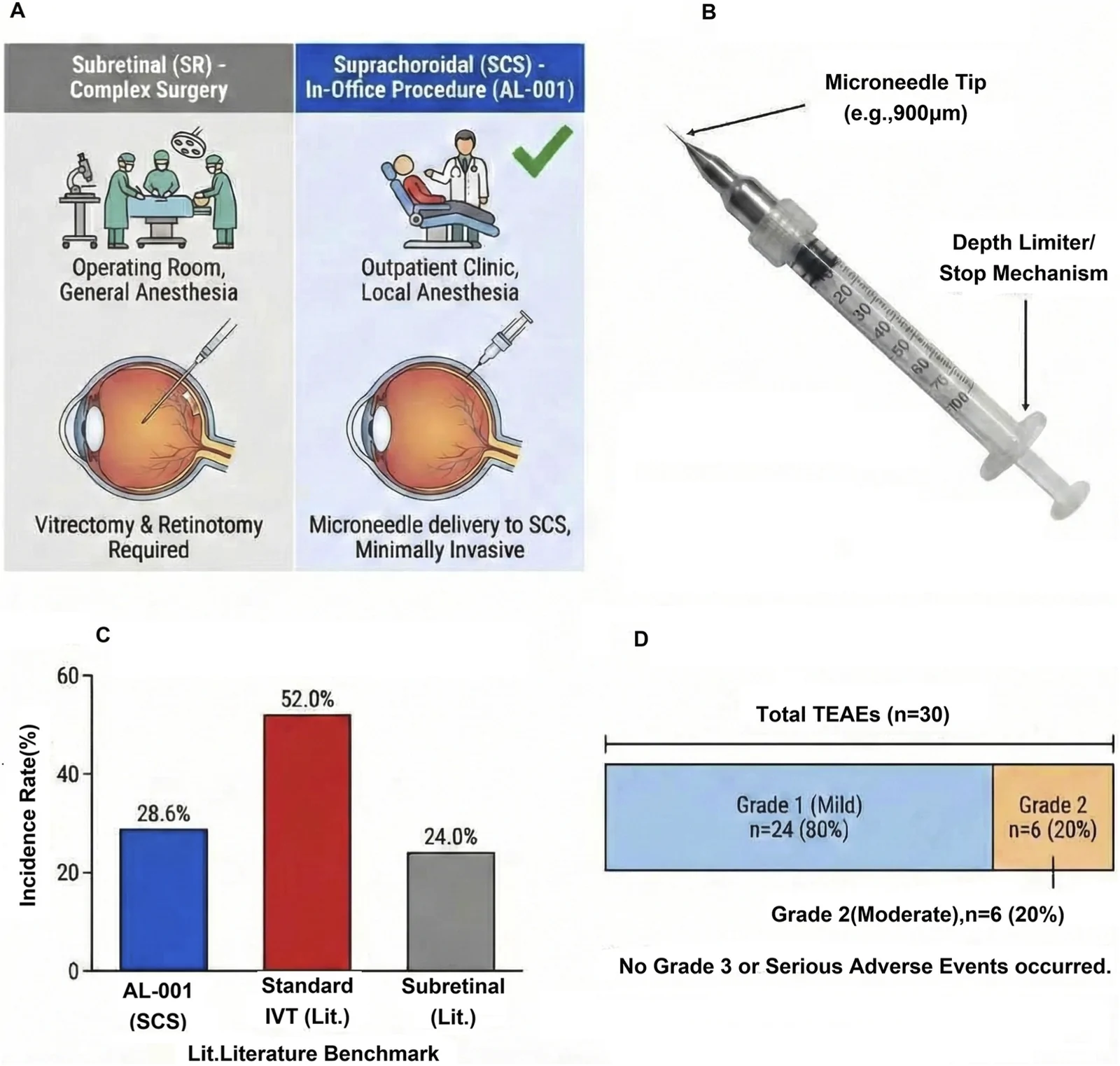
Suprachoroidal space (SCS) delivery approach and clinical safety profile of AL-001 **(A)** Schematic comparison of subretinal versus SCS drug delivery routes (created with BioRender.com) **(B)** Specialized micro-injector designed for precise SCS administration (38G, 900 μm depth-limited SCS delivery device; Beijing Medical Robots Industry Innovation Center, Beijing, China) **(C)** Comparative analysis of ocular inflammation incidence rates **(D)** Severity distribution of treatment emergent adverse events (TEAEs) in the safety analysis set (*N* = 7 subjects; *n* = 30 total events).

As illustrated in Figure 6A, subretinal administration requires vitrectomy and retinotomy performed in an operating room under general anesthesia, whereas SCS administration of AL-001 is conducted as an in-office procedure under local anesthesia using a microneedle-based injection. Drug delivery to the SCS was achieved using a dedicated micro-injector equipped with a microneedle tip and a depth-limiting mechanism to control the injection depth and facilitate targeted administration (Figure 6B).

Clinical safety was assessed by monitoring ocular inflammation and TEAEs. The incidence of ocular inflammation following SCS administration of AL-001 was 28.6% (2/7), which was within the range reported for subretinal delivery (24.0%) and lower than literature-reported rates for standard intravitreal injection (52.0%) (Figure 6C).

In the safety analysis set (*N* = 7), a total of 30 TEAEs was reported. Most events were classified as grade 1 (mild; 80.0%, 24/30), while the remaining events were grade 2 (moderate; 20.0%, 6/30). No grade 3 or serious AEs were observed during the study period (Figure 6D).

## Discussion

In this study, we comprehensively evaluated AL-001, a novel AAV-based gene therapy for choroidal neovascularization, and demonstrated that a single administration of AL-001 achieved efficient and sustained therapeutic protein expression in ocular tissues, resulting in efficient and durable suppression of CNV-associated vascular leakage in preclinical models. Through rational capsid and expression cassette engineering, AL-001 exhibited enhanced transduction efficiency in relevant ocular cells and appropriate anatomical localization *in vivo*. Notably, suprachoroidal delivery conferred a distinctly improved safety and biodistribution profile compared with intravitreal administration, characterized by reduced anterior segment exposure, lower inflammatory burden, and more anatomically confined vector distribution. This route-dependent safety advantage represents a critical translational strength of AL-001. Furthermore, integration of immunogenicity profiling with translational modeling offered preliminary insights into inter-individual anti-AAV immune responses and the potential feasibility of redosing. Collectively, these findings highlight the novelty of combining sustained antiangiogenic gene expression with a clinically practical suprachoroidal delivery strategy and support the translational potential of AL-001 and justify further clinical evaluation.

The efficient ocular expression observed with AL-001 is attributable to its rational capsid and expression cassette engineering. AAV2 was selected as the parental capsid because of its well-characterized retinal tropism, extensive preclinical and clinical experience in ocular gene therapy, and established manufacturability. Although AAV2 remains one of the most extensively characterized serotypes for ocular gene delivery, its transduction efficiency in posterior segment tissues can be limited depending on the delivery route and cell type (Koponen et al., 2023; Whitehead et al., 2023). In this study, site-directed evolution was applied to an AAV2-derived capsid, introducing defined point mutations at surface-exposed residues while preserving overall capsid architecture, as supported by structural modeling. This approach is consistent with recent reports demonstrating that modest capsid modifications can significantly enhance retinal tropism without compromising vector stability or manufacturability (Crosson et al., 2021; Whitehead et al., 2023). In the present study, the engineered capsid demonstrated efficient ocular transgene expression *in vitro* and *in vivo*. However, direct head-to-head comparisons with wild-type AAV2 or other serotypes such as AAV8 were not included in the current study and therefore warrant further investigation in future studies.

In parallel, the AL-001 expression cassette was optimized through the integration of a tissue-specific promoter, regulatory elements, and codon optimization to enhance transcriptional and translational efficiency. Recent studies have highlighted the importance of promoter selection and transgene optimization in achieving durable and cell-relevant ocular expression (Buck and Wijnholds, 2020; Nieuwenhuis et al., 2023). Consistent with this rationale, AL-001 demonstrated higher transgene expression in ARPE-19 cells than in HEK293 cells, supporting its capacity for transgene expression in ocular-relevant cells and providing supportive evidence for the *in vivo* expression observed following ocular administration.

Regarding the stability data presented in Table 2, it is important to clarify that the observed decline in biological activity at 6 months (to 45%) was observed under −20 °C storage conditions, which served as an early-stage formulation exploration to characterize vector sensitivity. AAV vectors are known to be susceptible to physical degradation, such as capsid disassembly or genome ejection, when stored at nonideal temperatures.

To address this, ongoing optimization of the formulation is focused on the incorporation of novel cryoprotectants, such as trehalose or sucrose, to preserve capsid stability and preserve biological activity during storage. In addition, for clinical and commercial applications, more stringent storage conditions (≤−60 °C) and/or the development of lyophilized formulations are being established to ensure long-term potency and a sustained shelf-life.

Using the laser-induced CNV model, a well-established platform for evaluating antiangiogenic therapies (Fabian-Jessing et al., 2022), AL-001 significantly reduced fluorescein leakage and lesion severity over multiple time points. The timing of CNV induction in this study was determined based on preliminary optimization experiments, aiming to align the peak phase of lesion development with the establishment of stable transgene expression. This design allows for a more accurate evaluation of the therapeutic effect under conditions of sustained intraocular protein expression. These results align with, but also extend, recent advances in AAV-mediated anti-VEGF gene therapy. Several programs, including RGX-314 and ADVM-022, have demonstrated the feasibility of sustained intraocular anti-VEGF expression following a single administration (Gelfman et al., 2021; Campochiaro et al., 2024). However, variability in durability, inflammatory responses, and delivery-related limitations remain active challenges in the field.

Compared with conventional intravitreal anti-VEGF biologics that require frequent dosing, gene therapy aims to provide long-term disease control with a reduced treatment burden (Rowe and Ciulla, 2024). The sustained suppression of CNV leakage observed in this study supports the potential of AL-001 to address unmet needs in chronic neovascular retinal diseases, including neovascular age-related macular degeneration and other CNV-related disorders.

A key innovation of this study is the use of SCS delivery for AAV-mediated gene therapy. Subretinal delivery, while effective, requires temporary focal retinal detachment and creation of a retinotomy; additionally, it is associated with surgical complexity and limited scalability (Kansara et al., 2020). Intravitreal delivery, although minimally invasive, can result in widespread ocular exposure and has been associated with dose-dependent inflammation in recent clinical studies (Liu et al., 2025). The SCS route offers an alternative delivery strategy that enables efficient drug access to the posterior segment while minimizing anterior segment exposure (Wu et al., 2023). The SCS provides a potential anatomical compartment between the sclera and choroid that enables targeted delivery to the posterior segment while limiting exposure to the vitreous and anterior segment (Kansara et al., 2020; Wu et al., 2023). Although the choroid is highly vascularized, raising theoretical concerns regarding systemic vector dissemination, our pharmacokinetic and biodistribution analyses demonstrated that viral genomes were predominantly confined to the injected eye, with only transient and low-level detection in peripheral blood and no significant accumulation in major organs. In the present study, SCS administration of AL-001 resulted in a more favorable ocular biodistribution profile, reduced severe CNV leakage grades, and sustained therapeutic benefit during the observation period compared with intravitreal injection. Importantly, these findings are specific to the AAV capsid and construct used in AL-001 and should not be generalized to all AAV vectors or delivery strategies. These findings suggest that SCS delivery may achieve localized transgene expression without increased systemic exposure and may offer advantages in local targeting and safety profile relative to intravitreal administration, supporting its potential as a promising strategy for next-generation retinal gene therapies.

Long-term safety is a central consideration for ocular gene therapy. In the present study, AL-001 was well tolerated, as evidenced by low-grade and transient ocular inflammation, stable intraocular pressure, minimal systemic exposure of the expressed therapeutic protein, and largely ocular-restricted viral biodistribution. These findings are consistent with recent ocular AAV gene therapy study reports demonstrating acceptable safety profiles for ocular AAV-based gene therapies (Liu et al., 2025). Intravitreal delivery is clinically convenient and widely adopted; however, accumulating evidence indicates that high-dose intravitreal AAV administration may trigger dose-dependent intraocular inflammation, potentially related to widespread vector exposure to anterior segment tissues and immune surveillance pathways (Mehta et al., 2021; Liu et al., 2025). In contrast, SCS administration of AL-001 resulted in more anatomically targeted posterior segment distribution with reduced anterior chamber exposure. This spatial confinement was associated with lower inflammatory signals, stable intraocular pressure, and the absence of progressive ocular toxicity during follow-up. Importantly, the improved safety profile was achieved without compromising therapeutic efficacy, as evidenced by sustained suppression of CNV leakage. These findings suggest that the delivery route is not merely a procedural variable but a key biological determinant of immune activation and ocular tolerability in gene therapy. Collectively, these results suggest that the favorable safety profile of AL-001 may be attributable to a combination of rational vector engineering and suprachoroidal delivery, which together may help mitigate immune-mediated adverse effects.

Pre-existing and treatment-induced anti-AAV NAbs remain a key challenge for clinical translation. Recent studies indicate that baseline anti-AAV2 seroprevalence varies across populations but is generally moderate in ophthalmic indications (Gehrke et al., 2022; Qin et al., 2024). Consistent with previous studies, the baseline NAb levels in the present study were low in nonhuman primates and clinical subjects, supporting patient eligibility for treatment. Following administration, the NAb titers increased with substantial inter-individual variability. Rather than solely describing these responses, we integrated immunogenicity data into a translational modeling framework to explore their potential implications for treatment durability and redosing feasibility. This approach aligns with emerging efforts to incorporate immune profiling into gene therapy development and may inform future clinical trial design (Yang et al., 2022).

Several limitations should be acknowledged. First, although the CNV models employed are well established, they do not fully recapitulate the complexity of human neovascular diseases. Second, the histological validation was limited by the absence of corresponding control images, which restricts the assessment of staining specificity and precise localization of transgene expression. Third, longer-term and larger-scale clinical studies will be required to confirm durability and safety. Future investigations should also explore dose optimization, immune stratification, and the feasibility of repeat administration. In addition, although limited clinical safety observations were included to support translational feasibility, the current study was not designed to evaluate clinical efficacy outcomes, which will require further investigation in larger prospective clinical studies.

## Conclusion

In conclusion, the combination of rational AAV engineering and suprachoroidal delivery provides not only sustained antiangiogenic efficacy but also a differentiated safety profile compared with intravitreal gene therapy approaches. This safety–efficacy balance may be particularly relevant for chronic retinal diseases requiring long-term intraocular protein expression and supports a clinically translatable gene therapy strategy for the long-term treatment of CNV and related neovascular retinal diseases.

## Data Availability

The raw data supporting the conclusions of this article will be made available by the authors, without undue reservation.
